# Advanced Sensors Technologies Applied in Mobile Robot

**DOI:** 10.3390/s23062958

**Published:** 2023-03-08

**Authors:** Gregor Klančar, Marija Seder, Sašo Blažič

**Affiliations:** 1 Faculty of Electrical Engineering, University of Ljubljana, Tržaška 25, 1000 Ljubljana, Slovenia; 2 Faculty of Electrical Engineering and Computing, University of Zagreb, Unska 3, 10000 Zagreb, Croatia

This special issue focuses on mobile robotic systems, where we are seeing a widespread increase in current applications as well as promising future applications enabled by the latest technologies in sensor development. Mobile robots are already making their way into our homes, modern manufacturing and warehousing systems are hard to imagine without driverless transport systems. Self-driving cars are already driving on normal roads, flying taxis are about to take off and offer new travel experiences and drones already have applications in delivery and remote sensing. There are also applications in agriculture, construction, medical care, surveillance, entertainment and other areas, some of which will also develop in unforeseen ways, all of which offer an emerging market with great potential. Advanced sensor technologies are critical in mobile robotics—a multidisciplinary field of research—to achieve automated or autonomous operation of mobile robots in these applications. They play a role in any navigation, motion control, action planning, decision making, environmental recognition, localisation, perception, object recognition, target tracking or object manipulation.

This special issue on advanced sensor technologies contains contributions on the latest developments in mobile robotic systems and related research. Various topics with different ideas and applications from mobile robotics have found their place in this special issue. They can be grouped into the three main areas of localisation and situational awareness, path planning and control algorithms. The three areas fit well with the Sense-Plan-Act architecture, which describes the most important basic activities required for the implementation of cognitive autonomous systems.

## 1. Sensing for Localisation

In [1], a novel method for calculating the odometry of a 3D LiDAR range image in real time is presented. Ego-motion is computed by iteratively imposing a coplanarity constraint between pairs of detected planner objects in the first step and their associated keypoints in the second step. In [2], humanoid robot control is reported using state-of-the-art motion capture systems in the high-frequency feedback control loop of humanoid robots. This can be an alternative in cases where state estimation is not reliable. Such external estimators can serve as a reference for the internal estimators, as presented in this work. Fingerprinting-based indoor 2D positioning method is proposed in [3], which utilizes the fusion of RSSI and magnetometer measurements. Autonomous navigation in mining tunnels based on artificial passive landmarks is addressed in [4]. The geometry has been optimized in order to ensure drift-free localization of mobile units equipped with LiDAR scanners. Computationally efficient high-level B-spline features extraction from 2D LiDAR is proposed in [5] with application to mapping problems. This work also provides a new benchmark for evaluating and comparing different feature generators.

## 2. Sensing for Situation Awareness

The identification and classification of attention deficit hyperactivity disorder (ADHD) in children is outlined in [6]. This is done through a game in which a mobile robot animates a child who must follow the robot’s path. Using five Azure Kinect units equipped with depth sensors, the recorded skeletal data is analysed and classified using deep neural networks to output a diagnosis while the child carelessly plays the game. Article [7] presents an innovative strategy for collecting dirt samples for cleaning robots by combining geometric feature extraction and swarm algorithms. This combined approach generates an efficient optimal path that covers all identified dirt locations for an efficient cleaning mission. In addition, article [8] provides an annotated comprehensive dataset for dirt analysis. Nine classes of common domestic dirt and a labelled dataset of 3000 microscope dirt images taken from a semi-domestic environment. In [9], an AI-assisted system for predictive maintenance of mobile cleaning robots is presented that uses vibration signals to detect performance degradation and operational safety issues. A four-layer 1D convolutional neural network framework was developed and trained on a dataset of vibration signals generated by a self-developed autonomous steam mopping robot with different levels of degradation and hazardous operating environments. In [10], an approach is implemented to enable a drone to autonomously clean insulators on a power line. The algorithm for detecting and tracking dirty insulators is implemented and a special cleaning hardware is developed. In [11], a framework for false ceiling deterioration detection and mapping using deep neural network based object detection algorithm and teleoperated robot is presented. The object detection algorithm was trained on our custom false ceiling damage detection dataset consisting of four classes: structural defects (spalling, cracks, pitted surfaces and water damage), HVAC system damage (corrosion, mould and pipe damage), electrical damage (frayed wires) and pest infestation (termites and rodents).

## 3. Path Planning

Complete coverage path planning algorithm that generates smooth paths based on clothoids that allow a non-holonomic mobile robot to move in optimal time while following the path is described in [12]. This algorithm significantly reduces the coverage time, path length and overlap area, and increases the coverage rate compared to state-of-the-art full coverage algorithms. In [13], a novel solution for a spline path of a 5th order Bézier curve is proposed to obtain smooth trajectory planning with minimum time for wheeled mobile robots. The proposed trajectory optimisation considers constraints on the environmental space and constraints on the velocity, acceleration and jerk. In [14], a smooth navigation function combining Dijkstra-based discrete static potential field evaluation with bilinear interpolation is proposed. Modifications of the bilinear interpolation method are proposed to make it applicable to path-planning applications. In [15], a method based on gait biomechanics is presented for short-term prediction of pedestrian trajectories for real-time applications. This method relies on a single biomechanical variable and has a low computational cost, making it a viable solution for implementation in low-cost wearable devices.

## 4. Control Algorithms

An adaptive manipulator prescribed performance tracking motion control with global finite-time stability guarantees is proposed in [16]. In [17], a visual servo control approach is presented that enables an unmanned aerial vehicle to land autonomously on a fast-moving platform of another vehicle. In [18], the modelling and control of a spherical robot are proposed and tested with different control strategies. The model and examples with different control scenarios are available online. In [19], a global navigation function for model predictive control (MPC) of autonomous mobile robots with application to warehouse automation is proposed. The navigation function is based on a potential field derived from an E* graph search algorithm on a discrete occupancy grid and by bicubic interpolation.

This special issue brings innovative ideas that apply sensor technologies in mobile robotics in their own way. New ideas are presented for mobile robots that specialise in cleaning floors, power lines and HVAC systems. We also find innovative approaches to navigation path planning using local-minima-free potential fields, novel path primitives and/or their parameterisation for minimum-time planning, and various control approaches ranging from visual servoing to model predictive and adaptive trajectory tracking, applied to wheeled robots, humanoid manipulators and flying robots. Localisation approaches using LiDAR, motion capture systems, fingerprint-based and biomechanical gait systems are also discussed. In addition to advances in methodology, applications in healthcare, mining tunnels, cleaning, warehouses and other areas are mentioned. We believe that the works collected in the special issue Advanced Sensors Technologies Applied in Mobile Robot and its results will inspire other researchers in solving future research questions and applications in mobile robotics.

## Data Availability

Not applicable.
